# Inhibition of EZH2 Ameliorates Sepsis Acute Lung Injury (SALI) and Non-Small-Cell Lung Cancer (NSCLC) Proliferation through the PD-L1 Pathway

**DOI:** 10.3390/cells11243958

**Published:** 2022-12-07

**Authors:** Ziyi Wang, Zhe Guo, Xuesong Wang, Haiyan Liao, Yan Chai, Ziwen Wang, Zhong Wang

**Affiliations:** School of Clinical Medicine, Tsinghua University, Beijing 100190, China

**Keywords:** SALI, NSCLC, EZH2, apoptosis, WGCNA

## Abstract

(1) Background: Both sepsis acute lung injury (SALI) and non-small-cell lung cancer (NSCLC) are life-threatening diseases caused by immune response disorders and inflammation, but the underlining linking mechanisms are still not clear. This study aimed to detect the shared gene signature and potential molecular process between SALI and NSCLC. (2) Methods: RNA sequences and patient information on sepsis and NSCLC were acquired from the Gene Expression Omnibus (GEO) database. Weighted gene co-expression network analysis (WGCNA) was used to build a co-expression network associated with sepsis and NSCLC. Protein–protein interaction (PPI) analysis of shared genes was intuitively performed by the Search Tool for the Retrieval of Interacting Genes/Proteins (STRING) database. The involvement of *EZH2* in the tumor immune microenvironment (TIME) and sepsis immune microenvironment (IME) was assessed by R software. Western blot, flow cytometry, and other in vitro assays were performed to further confirm the function and mechanism of *EZH2* in NSCLC and SALI. (3) Results: WGCNA recognized three major modules for sepsis and two major modules for NSCLC, and there were seven shared genes identified for the two diseases. Additionally, the hub gene *EZH2* was screened out. It was shown that EZH2 was closely related to the IME in the two diseases. In the validation assay, our data showed that EZH2 was expressed at a higher level in peripheral blood mononuclear cells (PBMCs) of septic patients than those of healthy donors (HDs), and EZH2 was also expressed at a higher level in lipopolysaccharide (LPS)-induced PBMCs and non-small cell lung cancer (A549) cells. EZH2 inhibitor (GSK343) downregulated the proliferation ability of A549 cells in a concentration-dependent manner, parallel with the decreased expression level of PD-L1. Similarly, GSK343 inhibited PD-L1 protein expression and downregulated the level of proinflammatory factors in LPS-induced PBMCs. In the co-culture system of PBMCs and human type II alveolar epithelial cells (ATIIs), the addition of GSK343 to PBMCs significantly downregulated the apoptosis of LPS-induced ATIIs. (4) Conclusions: This study illustrated that EZH2 inhibition could ameliorate A549 cell proliferation and LPS-induced ATII apoptosis in parallel with downregulation of PD-L1 protein expression, which provided new insights into molecular signaling networks involved in the pathogenetics of SALI and NSCLC.

## 1. Introduction

Sepsis is a common critical illness related to immune disorders, and the lung is one of the most susceptible organs. In the early stage of sepsis, neutrophils and peripheral blood monocytes leak from damaged vascular endothelial cells into the lung interstitium, accumulate around the alveoli, and release a number of proinflammatory factors. Excessive proinflammatory factors lead to alveolar epithelial cell injury, resulting in sepsis acute lung injury (SALI) [1]. If the outbreak of excessive inflammatory factors could be effectively blocked, we could prevent the progression of inflammation to irreversible organ function damage in sepsis [2]. Based on this, in 2020, Chinese emergency experts put forward ideas for preventing and blocking sepsis, emphasizing “early prevention, early detection, and early intervention” to reduce the incidence of sepsis and the mortality of infection [3]. Therefore, it is essential to investigate early biomarkers to identify individuals at a high risk of developing sepsis.

Non-small-cell lung cancer (NSCLC) accounts for about 85% of all lung cancers, and most patients with NSCLC are in the advanced stage at diagnosis [4]. The balance between anti-tumor immune cells and immunosuppressive cells in the tumor immune microenvironment (TIME) affects NSCLC genetics and development [5]. At present, chemotherapy is still the main treatment, and the overall prognosis is poor. Improving the treatment status and increasing long-term survival are the most urgent needs for advanced NSCLC patients. In recent years, tumor immunotherapy has developed rapidly, and immune checkpoint inhibitors (ICIs), especially ICIs targeting programmed death factor-1 (PD-1) and programmed death ligand-1(PD-L1), have made breakthrough progress in the treatment of NSCLC with negative driver gene mutations, bringing survival benefits to patients and changing the treatment pattern of NSCLC [6].

Since immune disorders play a key role in both SALI and NSCLC, we wondered whether ICIs for NSCLC could be used in SALI prevention and treatment. Although their shared signatures based on gene regulation mechanisms are still unknown, the rise of sequencing technology and bioinformatics in recent years has made it possible to explore the link between the two diseases at the genetic level [7,8]. The Gene Expression Omnibus (GEO) database was employed to obtain the RNA sequence and patient information regarding NSCLC and sepsis. Weighted gene co-expression network analysis (WGCNA) was used to identify the co-expression network linked with sepsis and NSCLC. A protein–protein interaction (PPI) network was utilized to investigate key shared genes. The levels of immune cells were compared by single-sample gene set enrichment analysis (ssGSEA) scores. Our study was designed to explore the link between the two diseases through bioinformatics and verified the hub genes via molecular biology experiments. It was demonstrated that seven shared genes might be considered the link between NSCLC and SALI. *EZH2*, as the key shared gene, was positively related to PD-L1 and might be used as the bridge for detecting the immune mechanism between SALI and NSCLC. Therefore, our study filled a research gap from both theoretical and empirical research perspectives.

## 2. Materials and Methods

### 2.1. Bioinformatics Analysis

#### 2.1.1. Dataset Download 

We employed the term “non-small lung cancer” or “sepsis” to retrieve NSCLC and septic patients’ gene expression profiles from the GEO database (http://www.ncbi.nlm.nih.gov/geo/ (accessed on 1 August 2022)). Two GEO datasets, GSE43458 and GSE96455, were chosen for discovering the shared genes. Immunohistochemical data were gained from the HPA (https://www.proteinatlas.org/ (accessed on 1 August 2022)). The correlation analysis between *EZH2* and immune and inflammation-related factors was obtained from the tumor immune estimation resource (TIMER, http://timer.compgenomics.org/ (accessed on 1 August 2022)) database.

#### 2.1.2. Weighted Gene Co-Expression Network Analysis

WGCNA is a systematic biological method used to describe gene association patterns between different samples. It can be used to identify highly synergistic gene sets and identify candidate biomarker genes or therapeutic targets based on the interconnectedness of gene sets and the association between gene sets and phenotypes. First, we built the adjacency matrix using soft threshold b and the gene–gene correlation matrix to describe the degree of association between the nodes. The adjacency matrix was then converted into the topological overlap matrix (TOM). Next, the gene hierarchical clustering dendrogram was used to identify co-expression modules. Finally, we calculated the module eigengene (ME), as well as the correlation between ME and clinical traits, to identify clinical-related modules.

#### 2.1.3. PPI Network Construction and Key Target Screening

The PPI network was constructed by inputting the common targets of drug and disease into the Search Tool for the Retrieval of Interacting Genes/Proteins (STRING) database (https://string-db.org/cgi/input.pl (accessed on 1 August 2022)), and the species was set as “*Homo sapiens*” to obtain the PPI network. Then, TSV files obtained from the STRING database were imported into Cytoscape software to conduct topological analysis of the PPI network and screen out key target genes of compounds acting on diseases according to degree value ranking.

#### 2.1.4. Assessment of the Immune Landscape

The TIME was assessed by the single sample Gene Set Enrichment Analysis (ssGSEA) method using the R package “GSVA”. ssGSEA, an extension of GSEA, calculated separate enrichment scores (ESs) for each sample. The levels of immune cells between high and low *EZH2* expression groups were then compared by ssGSEA scores [9].

### 2.2. Molecular Biological Verification

#### 2.2.1. Basic Information

This study was performed under ethical approval from Tsinghua Changgung Hospital (NCT05095324). Using Sepsis 3.0, 25 samples were gathered at Beijing Tsinghua Changgung Hospital from 1 April 2022 to 1 July 2022, including 15 septic patients and 10 healthy donors. Peripheral blood samples were collected in EDTA tubes. All experiments met current standards. Table 1 shows the clinical characteristics of the study population.

#### 2.2.2. Entry and Exclusion Criteria 

The inclusion criteria included (1) patients meeting the diagnostic criteria for sepsis [10]; (2) patients with acute lung injury who met the relevant diagnostic criteria in the Diagnosis and Treatment of Acute Lung Injury/Acute Respiratory Distress Syndrome (2006) [11]; and (3) patients with complete clinical data. The exclusion criteria were (1) severe liver and kidney insufficiency or failure; (2) immune system-related diseases without systematic treatment; (3) the use of hormones or immunosuppressants in the past 3 months; (4) a history of malignant tumors; and (5) unwillingness to participate or cooperate.

#### 2.2.3. Cell Culture

A549 cells were purchased from Wuhan Pricella Life Technology Co., LTD. (Wuhan, China) PBMCs and ATIIs were purchased from Hunan Fenghui Biotechnology Co., LTD. (Changsha, China) PBMCs and ATIIs were cultured in RPMI 1640 (Solarbio, Beijing, China), and A549 cells were cultured in Dulbecco’s Modified Eagle Medium (DMEM) containing high glucose (Solarbio, Beijing, China). The cells were cultured in an Incubator (SANYO, Osaka, Japan) under standard conditions (37 °C, 5% CO_2_) at the laboratory of Tsinghua Changgung Hospital. The experiments were performed after two passages. 

#### 2.2.4. Drug

GSK343, obtained from Shanghai Topscience Limited Corporation (Shanghai, China), was used as an EZH2 inhibitor. The powder was stored at −20 °C and was dissolved in DMSO and then adjusted to pH 4.5 with 1 N acetic acid for in vitro studies before use.

#### 2.2.5. Reagents

BioSci™ New Flash Protein any KD PAGE was obtained from Dakewe (Shenzhen, China). Anti-mouse IgG second antibody, goat anti-rabbit second antibody, anti-EZH2, and anti-PD-L1 antibody were acquired from Abcam (Cambridge, UK). The Annexin V -FITC/PI Apoptosis Kit was purchased from Solarbio (Beijing, China). Ten-percent fetal bovine serum (FBS) and crystal violet were obtained from Beyotime (Nantong, China).

#### 2.2.6. Western Blot

Total protein was extracted from cells using the cell lysate and placed on a shaker for 15 min for full contact at 4 °C. After centrifugation at 12,000 r·min^−1^ for 15 min (radius 12 cm) at 4 °C, the supernatant was collected, and the protein concentration was determined by the BCA method. A protein sample (20 μg) was used for electrocoagulation with sodium dodecanylsulfonate (SDS) polyacrylamide and electrophoresis on a polyvinylidene fluoride membrane. Then, the membrane was blocked in 5% dried milk at 4 °C overnight. Next, the membrane was incubated with primary antibody at 4 °C overnight, followed by incubation with goat anti-mouse IgG secondary antibody for luminescence.

#### 2.2.7. Cell Counting Kit-8 

Cell counting kit-8 (CCK-8) was used to confirm sufficient concentrations and time points of LPS challenge. The cells were seeded at 2 × 10^3^ cells per well in 96-well plates overnight, and cell growth was then measured at various concentrations and time points using the CCK-8 kit (C0038, Beyotime, China) at 450 nm.

#### 2.2.8. Colony Formation

Colony formation was used to evaluate A549 cells’ proliferative abilities. About 1 × 10^3^ cells were placed into each well in 6-well plates. The growth medium was replaced every 3 days. After 7 days, the cells were fixed with 4% methanol, then stained with crystal violet, and finally photographed and counted under an inverted microscope.

#### 2.2.9. Transwell Co-Culture Assay

Transwells with 0.4 μm pore size (Corning, New York, NY, USA) were used for non-contact co-culture of two types of cells. A total of 2 × 10^4^ PBMCs were pretreated with LPS and/or GSK343 for 12 h. The GSK343 group and the LPS + GSK343 group were treated with GSK343 for 1 h in advance. Then, the upper chambers with 2 × 10^4^ PBMCs and the lower chamber with 2 × 10^4^ ATIIs were co-cultured for 12 h in a 24-well plate. Both were supplemented with RPMI-1640 containing 10% FBS. PBMCs were kept in the upper chamber, and 2 × 10^4^ ATIIs were kept in the lower chamber. The LPS and LPS + GSK343 groups were given 100 ng/mL of LPS, while the control and GSK343 groups were not given any stimulation. After 12 h, the upper chambers were removed, and flow cytometry analysis was performed for ATIIs in the lower chamber.

#### 2.2.10. Flow Cytometry Analysis

Flow cytometry was used for apoptosis detection. After 12 h of co-culture with PBMCs, ATIIs, which were plated into the lower chamber, were collected and incubated to assess the cell apoptosis level with flow cytometry. ATIIs were washed twice with phosphate-buffered saline (PBS) and resuspended in 100 µL of 1 × binding buffer mixed with 2.5 µL of Annexin-V–FITC and 2.5 µL of 7-AAD staining solution for 15 min in the dark at room temperature. Then, with 400 µL of additional binding buffer added into the mix, the cells were finally evaluated using a flow cytometer (BD, Franklin Lakes, NJ, USA) for apoptosis. Flow cytometric analyses were performed using the Annexin V/7-ADD staining method to assess the possible protective effects of EZH2 inhibitor against LPS-induced ATII apoptosis. In brief, ATIIs were collected from the lower chamber of the co-culture Transwell, as stated in the previous step. After washing the treated cells in cold PBS, they were suspended in 100 μL of ice-cold binding buffer and stained with Annexin V/7-AAD at room temperature in the darkness. Annexin-V and 7-AAD assay Q2 (early apoptosis) + Q3 (late apoptosis) were used to determine the total apoptosis rate.

### 2.3. Data Processing and Statistical Analysis

Data were expressed as the mean ± standard deviation (SD). The differences between variables were compared using one-way ANOVA or by a two-tailed Student’s *t*-test using SPSS software (version 22.0; Chicago, IL, USA). Statistical significance was set at *p* < 0.05. Prism 8 (GraphPad Software, San Diego, CA, USA) was responsible for visualization.

## 3. Results

### 3.1. Identification of a Shared Molecular Connection between SALI and NSCLC

**Figure 1:**
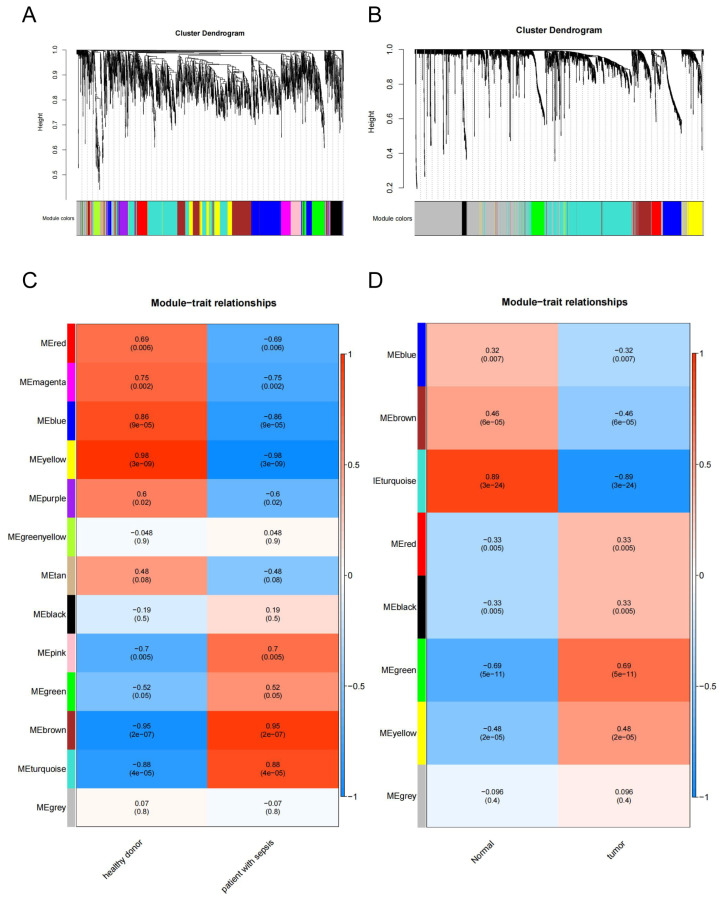

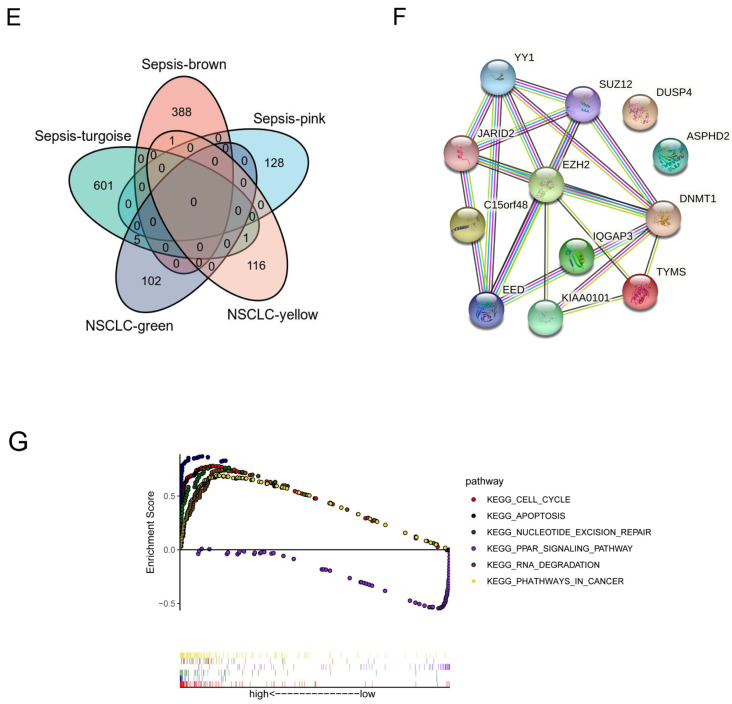
Identification of modules linked to clinical features of SALI and NSCLC. (**A**,**B**) Cluster dendrogram of co-expressed genes in SALI (**A**) and NSCLC (**B**). (**C**,**D**) Heat map of module–trait relationships in SALI (**C**) and NSCLC (**D**). (**E**) Venn diagram of the shared genes between the SALI modules and NSCLC modules. (**F**) PPI network of shared genes. (**G**) GSEA of the top 6 enriched pathways in sepsis patients with high EZH2 expression. GSEA, Gene Set Enrichment Analysis.

To identify the shared molecular connection between SALI and NSCLC, a heat map of module–trait relationships was drawn to assess the relationships between modules based on the Spearman correlation coefficient (Figure 1A,C). There were 13 modules discovered in GSE46955. Three, “pink,” “brown,” and “turquoise”, modules had a highly positive association with sepsis and were chosen as sepsis-related modules (pink module: r = 0.7, *p* = 0.005, genes = 128; brown module: r = 0.95, *p* = 2 × 10^−7^, genes = 389; turquoise module: r = 0.88, *p* = 4 × 10^−5^, genes = 607). Additionally, eight modules were discovered in GSE43458, with the module “green” (r = 0.69, *p* = 5 × 10^−11^, genes = 107) and “yellow” (r = 0.48, *p* = 2 × 10^−5^, genes = 118) being highly positively linked with NSCLC (Figure 1B,D). Sepsis and NSCLC shared seven genes in positively related modules (Figure 1E), which were considered to be significantly associated with the pathogenesis of sepsis and NSCLC. 

To determine the hub gene, we continued to construct the PPI network at the protein level from shared modules (Figure 1F). Because the number of molecules was limited, we used an enlarged PPI network, which showed that *EZH2* was most associated with other shared molecules. Enrichment analysis suggested that these shared molecules were mostly involved in pathways of the cell cycle, apoptosis, nucleotide excision repair, PPAR signaling, DNA degradation, and cancer (Figure 1G).

### 3.2. Diagnostic and Prognostic Value of the Hub Gene EZH2

**Figure 2:**
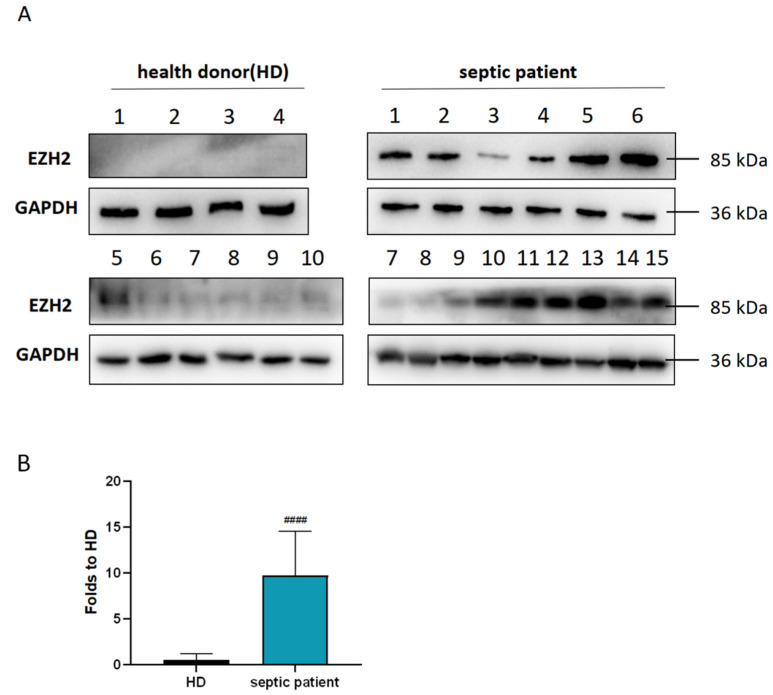

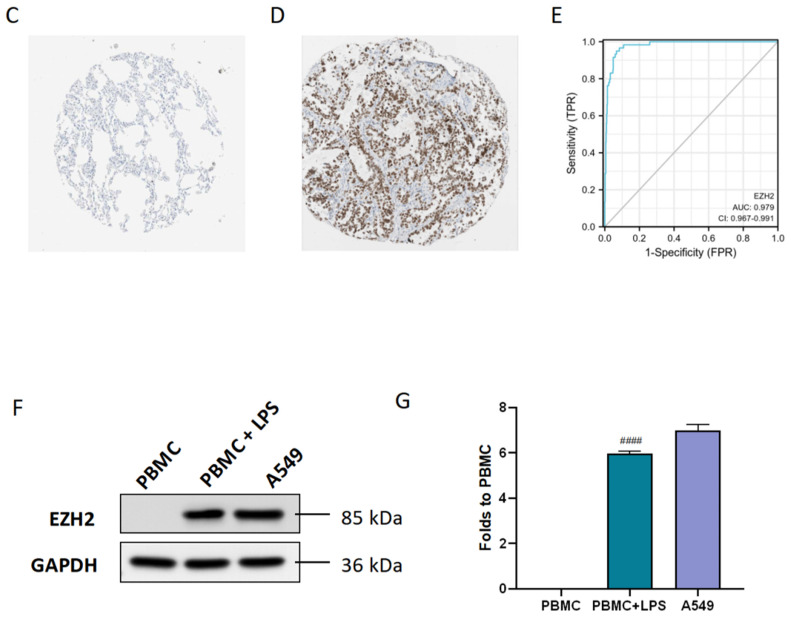
EZH2 could be a potential biomarker for SALI and NSCLC. (**A**,**B**) Western blot assay was performed to assess the EZH2 expression level in healthy donors (HDs) and septic patients. The fold expression was measured by densitometric analysis. (**C**,**D**) Representative IHC staining of EZH2 in normal and NSCLC tissues from the HPA database. (**E**) ROC curve of EZH2 in NSCLC patients from the TCGA database. (**F**,**G**) Western blot assay was performed to assess the EZH2 expression level in LPS-induced PBMCs and A549. The fold expression was measured by densitometric analysis. Values are expressed as mean ± SD. ^####^ *p* < 0.001, relative to the control group, *n* = 3.

To detect the diagnostic and prognostic value of EZH2 in SALI, clinical data from Tsinghua Changgung Hospital were collected. As shown in Table 1, there were no significant differences in gender, age, body mass index (BMI), and primary disease between the healthy donors (HDs) group and the SALI group (*p* > 0.05). Western blot results (Figure 2A,B) showed that the expression level of the EZH2 protein in the SALI group was significantly higher than that in the HD group (*p* < 0.005). The average WB gray value was taken as the boundary, the high group was classified as the high EZH2 group, and the low group was classified as the low EZH2 group. The length of hospital stay in the low EZH2 group was significantly shorter than that in the high EZH2 group, and the oxygenation index in the low EZH2 group was significantly higher than that in the high EZH2 group, with statistically significant differences (*p*< 0.05). Immunohistochemical results of EZH2 in normal lung tissues and NSCLC lung tissues were obtained from the Human Protein Atlas (HPA) database (Figure 2C,D). EZH2 was almost not expressed in normal lung tissues but was highly expressed in NSCLC lung tissues. According to the receiver operating characteristic (ROC) curve obtained from the Cancer Genome Atlas (TCGA) database (Figure 2E), the area under the ROC curve (AUC) was as high as 0.979, indicating that EZH2 had high sensitivity and specificity for the diagnosis of NSCLC. As shown in Figure 2F, the expression of EZH2 in lipopolysaccharide (LPS)-induced peripheral blood mononuclear cells (PBMCs) was higher than that in the control group, and the expression of EZH2 was also higher in NSCLC (A549) cells.

**Table 1:** The clinical characteristics of the study population.

	HD	Septic Patient		*p*-Value
		Low EZH2	High EZH2	
Basic Information				
Patients (number of patients)	10	7	8	-
Gender: number of male patients (% within gender)	5(50%)	3(42.86%)	3(37.5%)	0.866
Age range	30–86	44–94	49–84	-
BMI	23.23 ± 2.32	23.63 ± 2.37	23.81 ± 2.01	0.8560
Age (*Mean ± SD*)	56.1 ± 20.89	71.71 ± 12.55	72.38 ± 14.94	0.0955
Protopathy disease [*n* (%)]				
Respiratory diseases	-	0.75	0.6	0.833
Digestive diseases	-	0.25	0.4	-
Hospital day, HOD (*Mean ± SD*)	-	59.71 ± 24.11	98.38 ± 34.16	0.0269
PaO_2_/FiO_2_, P/F (*Mean ± SD*)	-	281.6 ± 49.3	198.5 ± 42.17	0.0038

### 3.3. Association between EZH2 and the IME

**Figure 3:**
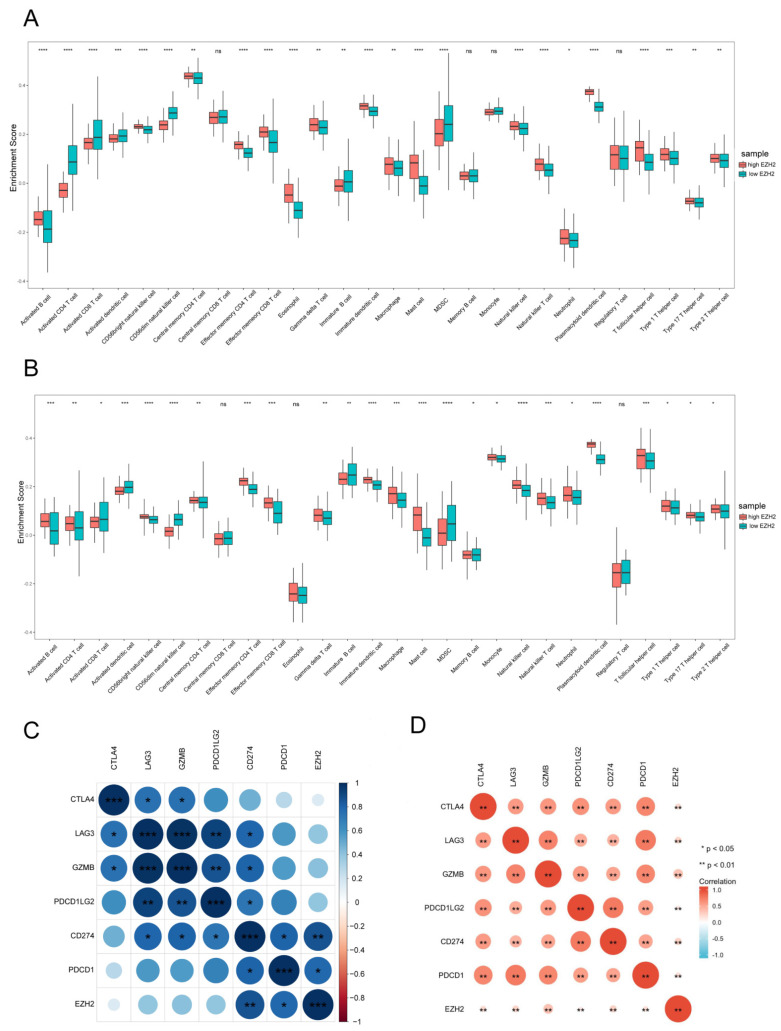
Characterization of the shared gene *EZH2* in immune cells. Comparison of immune cell subsets with high *EZH2* expression and low *EZH2* expression in sepsis patients’ PBMCs (**A**) and NSCLC patients’ lung tissues (**B**). Spearman correlation analysis of *EZH2* and T cell exhaustion-related molecules in sepsis (**C**) and NSCLC (**D**). Markers include *CD274*, *PDCD1*, *CTLA4*, *LAG3*, *GZMB*, and *PDCD1LG2* of T cell exhaustion. * *p* < 0.05, ** *p* < 0.01, *** *p* < 0.005, **** *p* < 0.001, ns: no significance.

To illustrate the association between *EZH2* and the IME, we conducted a study on the correlation between *EZH2* and immune cells to explore the role of *EZH2* in sepsis and the NSCLC immune landscape. Based on the ssGSEA algorithm, we compared the TIME landscape between the *EZH2*-high and *EZH2*-low groups (Figure 3A). Additionally, *EZH2* was shown to be positively linked with macrophages, natural killer (NK) cells, neutrophils, T helper 2 (Th1) cells, T helper 2 (Th2) cells, and T helper 17 (Th17) cells, while being negatively correlated with regulatory T (Treg) cells. We also compared the IME landscape of sepsis between the *EZH2*-high and *EZH2*-low groups (Figure 3B). *EZH2* was shown to be positively linked with macrophages, NK cells, Th1 cells, Th2 cells, Th17 cells, neutrophils, and DC cells, while being negatively correlated with B cells, clusters of differentiation (CD) 8 T cells, and CD4 T cells.

It was demonstrated that there was a molecular correlation between *EZH2* and T cell depletion-related factors. As shown in Figure 3C, *EZH2* was positively correlated with *CD274* (PD-L1) and *PDCD1* (PD-1) expression in sepsis, with statistical significance. As shown in Figure 3D, *EZH2* was positively correlated with *CD274* (PD-L1), *PDCD1* (PD-1), *CTLA4*, *LAG3*, *GZMB*, and *PDCD1LG2* (PD-L2) in NSCLC, showing statistical significance. *EZH2* was positively correlated with *CD274* (PD-L1) in both sepsis and NSCLC, and the correlation was statistically significant.

### 3.4. Identification of Sufficient Concentrations and Time Points of LPS Challenge

**Figure 4:**
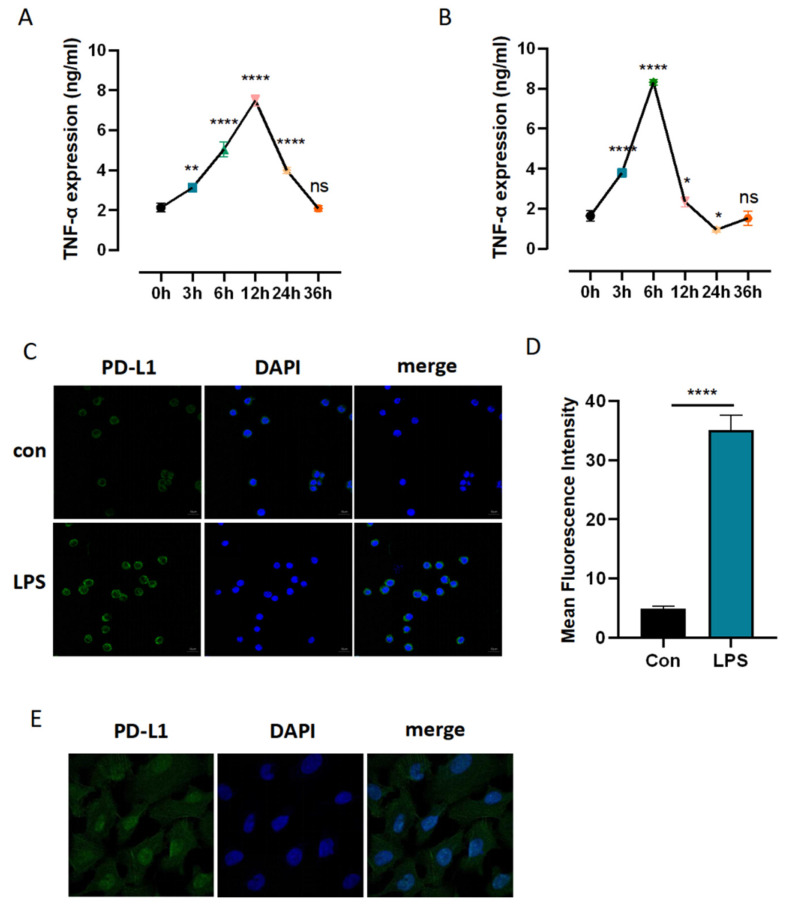

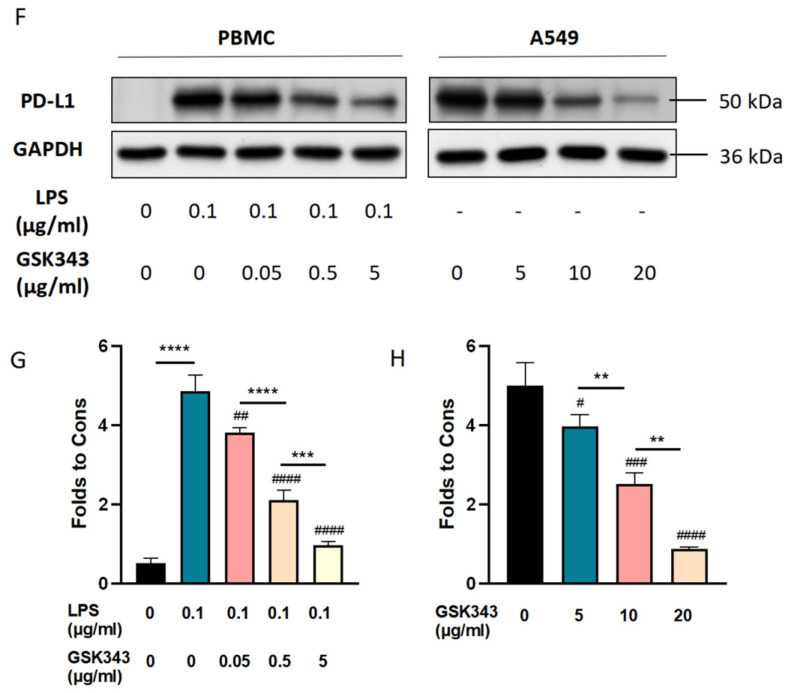
GSK343 decreased the protein level of PD-L1 in LPS-induced PBMCs and A549. (**A**) The effect of 100 ng/mL of LPS on the expression of TNF-α in PBMCs. (**B**) The effect of 1 μg/mL of LPS on the expression of TNF-α in PBMCs. (**C**) Representative IHC staining of EZH2 in normal and NSCLC tissues from the HPA database. (**D**,**E**) Immunofluorescence was performed to detect the protein expression level of PD-L1. Magnification ×20, scale bar 50 μm. (**F**–**H**) The protein expression levels of PD-L1 in LPS-induced PBMCs and A549 were analyzed by Western blotting. The results were quantified by densitometry. The fold expression was measured by densitometric analysis. Data are presented as mean ± SD (*n* = 3 per group) of the representative data from three independent experiments; * *p* < 0.05, ** *p* < 0.01, *** *p* < 0.005, **** *p* < 0.001, ^#^ *p* < 0.05, ^##^ *p* < 0.01, ^###^ *p* < 0.005, ^####^ *p* < 0.001, ns: no significance. The symbol (#) shows that the group is statistically different from the Con group.

To identify the sufficient concentrations and time points of LPS challenge for the following validation experiments, the level of tumor necrosis factor (TNF)-α was determined by enzyme-linked immunosorbent assay (ELISA). As shown in Figure 4A,B, the LPS group was treated with different concentrations of LPS (100 ng/mL and 1 μg/mL) for 3 h, 6 h, 12 h, 24 h, and 36 h. The control (Con) group was not treated with this intervention. The level of TNF-α was consistently enhanced when LPS was administrated at 100 ng/mL for 3 h and 6 h and peaked at 12 h. The level of TNF-α was increased when LPS was administrated at 1 μg/mL for 3 h and peaked at 6 h but hit a low point at 24 h. We selected 100 ng/mL LPS treatment for 12 h for the following experiment.

### 3.5. Inhibition of *EZH2* Suppressed PD-L1 Expression in LPS-Induced PBMCs and A549

To further explore how EZH2 regulates immune and inflammatory responses and plays a role in the occurrence and development of SALI, we took the correlation between EZH2 and PD-L1 as an entry point. We found that the expression of PD-L1 in PBMCs of the sepsis cell model constructed by LPS stimulation was higher than that in PBMCs of the control group (Figure 4C,D). EZH2 inhibitor GSK343 was used as the intervention method, and the cells were divided into five groups: a control group, an LPS group, and three LPS + GSK343 groups (0.05 μg/mL, 0.5 μg/mL, and 5 μg/mL). GSK343 was added one hour before LPS. As shown in Figure 4F,G, compared with the Con group, the protein expression level of PD-L1 was critically enhanced in the LPS group (*p* < 0.005). Compared with the LPS group, the protein expression level of PD-L1 was decreased in the LPS + GSK343 group, and the PD-L1 expression level was decreased with the increase in GSK343 concentration, with a statistically significant difference (*p* < 0.05).

Then, we explored the relationship between EZH2 and PD-L1 in NSCLC development. Through the HPA database, we found that the expression of PD-L1 in NSCLC lung tissues was higher than that in normal lung tissues (Figure 4E). A549 cells were divided into the control and GSK343 groups (5 μg/mL, 10 μg/mL, and 20 μg/mL), with EZH2 inhibitor GSK343 as the intervention method. As shown in Figure 4F,H, compared with the control group, the protein expression level of PD-L1 was significantly reduced in the GSK343 group (*p* < 0.005). Compared with the LPS group, the protein expression level of PD-L1 was decreased in the GSK343 group, and the expression level of PD-L1 was decreased with the increase in GSK343 concentration. The difference was statistically significant (*p* < 0.05).

### 3.6. Inhibition of EZH2 Ameliorated A549 Proliferation

**Figure 5:**
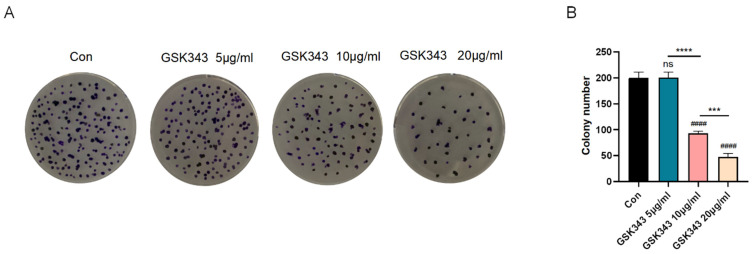
GSK343 alleviated A549 proliferation. (**A**) Representative images of colony formation and cell migration. (**B**) Inhibition of EZH2 significantly reduced colony formation ability in a concentration-dependent way. Data are presented as mean ± SD (*n* = 3 per group) of the representative data from three independent experiments; *** *p* < 0.005, **** *p* < 0.001, ^####^ *p* < 0.001, ns: no significance. The asterisk (*) demonstrates that the group is statistically different from the Con group.

To clarify the protective effect of GSK343 in NSCLC, colony formation assays were used to exhibit A549 proliferation. As shown in Figure 5A,B, a Con group and three GSK343 groups were inoculated in 6-well plates, cultured for 7 days, stained with 0.1% crystal violet, and photographed to compare the formation of clones in each group. The number of cell clones formed in the 5 μg/mL GSK343 group was not significantly different from that in the Con group, while the number of cell clones formed in the 10 μg/mL GSK343 and 20 μg/mL GSK343 groups was significantly less than that in the Con group. The number of cell clones formed in the 20 μg/mL GSK343 group was lower than that in the 10 μg/mL GSK343 group. In conclusion, inhibition of EZH2 greatly suppressed migratory cell abilities in a concentration-dependent manner.

### 3.7. Inhibition of EZH2 Ameliorated LPS-Induced PBMC Apoptosis

**Figure 6:**
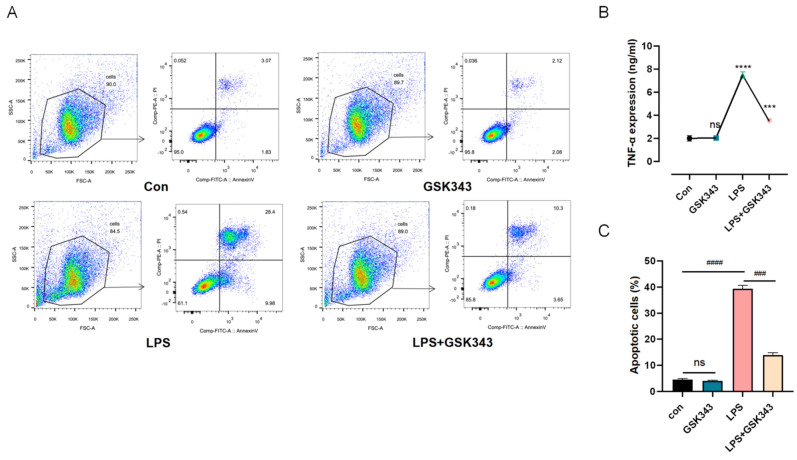
GSK343 alleviated LPS-induced ATII apoptosis. (**A**,**C**) Flow cytometry was employed to detect the apoptosis rate of ATIIs. The cells were equally divided into the Con group, LPS group, GSK343 group, and LPS + GSK343 group. We evaluated the proportion of apoptotic cells (Q2 + Q3) in each group. (**B**) The effect of GSK343 on the expression of TNF-α in LPS-induced PBMCs. Data are presented as mean ± SD (*n* = 3 per group) of the representative data from three independent experiments; *** *p* < 0.005, **** *p* < 0.001, ns: no significance, ### *p* < 0.005, #### *p* < 0.001. The asterisk (*) shows that the group is statistically different from the Con group.

To elucidate the protective effect of GSK343 in a closer pathological setting, we co-cultured PBMCs and ATIIs for 24 h using Transwell chambers. PBMCs were placed in the upper chamber. According to the treatment conditions, they were divided into the control group, LPS (1 μg/mL) group, LPS + GSK343 (0.05 μg/mL) group, LPS + GSK343 (0.5 μg/mL) group, and LPS + GSK343 (5 μg/mL) group. GSK343 was added one hour before the LPS addition. ATIIs were treated by being seeded in lower chambers with LPS. The apoptosis of ATIIs in the lower compartment was observed by flow cytometry. Annexin-V and 7-AAD assay Q2 + Q3 were used to exhibit the apoptosis rate. As shown in Figure 6A,C, LPS could significantly increase the apoptosis level (*p* < 0.001), GSK343 could critically alleviate it (*p* < 0.001), and the proportions of apoptosis cells were 4.46 ± 0.46, 4.03 ± 0.22, 39.40 ± 1.18, and 13.94 ± 0.94 in the Con group, GSK343 group, LPS group, and LPS + GSK343 group, respectively. The content of proinflammatory factor TNF-α in the supernatant of the upper chamber PBMC was detected by ELISA. As shown in Figure 6B, the level of TNF-α in the LPS group was slightly higher than that in the Con group when LPS was administrated at 100 ng/mL (*p* < 0.05). The level of TNF-α in the LPS + GSK343 group was significantly lower than that in the Con group (*p* < 0.05). There was no statistically significant difference between the GSK343 and Con groups.

## 4. Discussion

SALI and NSCLC, as diseases with high morbidity and mortality, are closely related to the inflammatory response and immune dysregulation. The IME plays an important role in NSCLC and SALI occurrence and development, but the link between them is still poorly understood. In this study, we analyzed the positive expression modules related to the two diseases using bioinformatics and found the molecules positively related to both diseases. Then, seven shared genes were considered for the link between NSCLC and sepsis. *EZH2* was selected as a key shared gene through the PPI network, which was positively linked to several immune-related molecular pathways in both sepsis and NSCLC. 

EZH2 is regarded as an important epigenetic regulator and one of two essential catalytic enzymes for histone H3 lysine 27 (H3K27) methylation in mammalian cells [12]. EZH2 also has the non-H3K27Me3-dependent function, which can directly mediate DNA methylation and methylation signal transduction [13,14]. EZH2 can directly bind DNA to achieve transcriptional activation. For example, in breast cancer cells, free EZH2 can directly bind the promoter of NOTCH1 to increase NOTCH1 expression [15]. This also provides evidence for our conjecture that EZH2 binds to the promoter of *CD274* (PD-L1) to increase PD-L1 expression, but this hypothesis still needs to be confirmed by chromatin immunoprecipitation-sequencing (ChIP-seq).

EZH2 was shown to be highly expressed in peripheral blood macrophages in a septic state, which was in parallel with our bioinformatics results [16]. The specific inhibitor of EZH2 can significantly inhibit the M1 differentiation of peritoneal macrophages, thereby reducing the inflammatory response in SALI [17,18]. The expressions of EZH2 in peripheral blood T lymphocytes and B lymphocytes of septic patients were both related to the severity of the disease, and the higher the expression of EZH2, the more severe the disease of septic patients [19,20,21,22,23]. Additionally, the sensitivity and specificity of EZH2 in the diagnosis of sepsis were higher than those of the APACHE II score and SOFA score. Our experiment confirmed that septic patients expressed more EZH2 in PBMCs than HDs, and patients with high EZH2 expression had a lower oxygenation index and longer hospital stays than patients with low EZH2 expression. Hence, patients with high expression of EZH2 in PBMCs had worse prognoses. 

Moreover, we continued to investigate LPS-induced EZH2 expression in vitro. We stimulated PBMCs with LPS to simulate the state of sepsis. TNF-α is a proinflammatory cytokine produced primarily by macrophages and monocytes. The changes in TNF-α levels in the supernatant were observed to determine the concentration and time of LPS stimulation. When PBMCs were treated with 100 ng/mL of LPS for 12 h and 1 μg/mL of LPS for 6 h, the level of TNF-α reached its peak. When PBMCs were treated with 1 μg/mL of LPS for 12 h, the level of proinflammatory factors reached the immunosuppressive state with a lower level of TNF-α at 36 h. This experiment mainly aimed to explore the state of inflammatory factors’ cascade outbreak; therefore, 100 ng/mL LPS administration for 12 h was selected for further investigation. Our results demonstrated that 100 ng/mL of LPS induced EZH2 protein expression in PBMCs.

As we know, sepsis is caused by an immune reaction disorder [24]. The most important pathological change of SALI is diffuse alveolar epithelial injury. The accumulation of immune cells is the key to alveolar epithelial cell damage. Under pathological conditions, macrophages and neutrophils secrete many inflammatory factors to promote inflammation and produce a cascade reaction to expand the inflammatory effect [25]. Similarly, several immune cells and tumor cells interact with various cytokines and chemokines to form the TIME, which plays an essential role in the development of NSCLC [26,27,28]. Therefore, it is of great significance to focus on the regulatory function of EZH2 in different immune cell types to guide clinical immunotherapy.

We further explored the relationship between *EZH2* and immune cells and immune-related factors. A series of reports have shown that our understanding of the role of *EZH2* in the TIME has been continuously expanded, including maintaining chronic inflammation and establishing immune tolerance, immune maintenance, and immune selection [29,30]. For example, EZH2 was shown to contribute to glioblastoma-induced immune deficiency by exacerbating inflammation, and inhibition of EZH2 could reshape the immune function of microglia [31]. EZH2 could also restrain the anti-tumor effect of macrophages and NK cells by suppressing the chemotaxis ability of tumors by reducing the expression level of CXCL10, CCL2, and other chemokines [32,33]. *EZH2* is prone to mutations in diffuse large B-cell lymphoma, which leads to acquired major histocompatibility complex (MHC)-II defects and tumor immune escape [34]. In our study, the level of *EZH2* was positively related to macrophages, NK cells, neutrophils, Th1, Th2, Th17, and *CD274* (PD-L1) in both NSCLC and sepsis datasets.

EZH2 and PD-L1 are both immune checkpoints, which have diverse and complex effects on the occurrence and development of cancer. As one of the possible immunotherapy-sensitizing drugs, an EZH2 inhibitor has been used in clinical trials of combination therapy in NSCLC, and the clinical effect has been promising [35]. However, there are still few studies on the relationship between EZH2 and PD-L1. Our study confirmed that the EZH2 inhibitor GSK343 downregulated the proliferation ability of A549 cells in a concentration-dependent manner, in parallel with the decreased expression level of PD-L1. In recent years, more attention has been paid to the relationship between PD-L1 and immune dysregulation in sepsis. Various immune cells have been found to highly express the PD-L1 protein in sepsis. Previous studies have mostly focused on the role of PD-L1 and immunosuppression in sepsis, but current studies have gradually found a link between PD-L1 and the hyperinflammatory response. For example, high expression of PD-L1 under a sepsis state could downregulate neutrophil apoptosis through the activation of the PI3K-AKT pathway, leading to the accumulation of neutrophils around the alveolar area and further aggravation of SALI [36]. In our study, EZH2 expression in LPS-induced PBMCs was higher than that in the control group, and PD-L1 expression decreased with the increase in GSK343 concentration. We used the Transwell assay to co-culture pretreated PBMCs with LPS-induced ATIIs to further simulate the physiological environment and to observe the effect of inhibition of EZH2 on ATIIs. In the co-culture system of PBMC and ATIIs, the addition of LPS + GSK343 to PBMCs (upper chamber) significantly downregulated the apoptosis of LPS-induced ATIIs (lower chamber). In other words, inhibition of EZH2 in PBMCs could alleviate alveolar epithelial cell injury at SALI. This provides the theoretical basis for the treatment of SALI with EZH2 inhibitors. 

This study revealed the significant role of *EZH2* in maintaining the IME and also suggested that NSCLC and SALI share some similarities in pathogenesis and treatment, which inspired us in terms of the prevention and treatment of sepsis. Thus, the current application of tumor immune checkpoint inhibitors may also play a critical role in improving the dysregulation of the immune response in sepsis.

## 5. Conclusions

**Figure 7:**
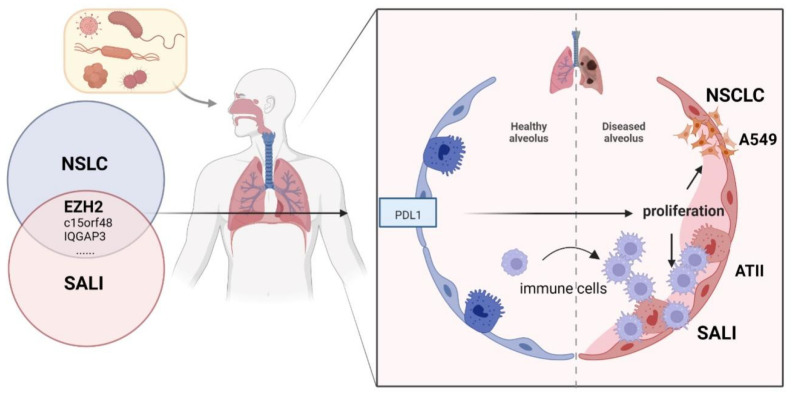
Overview of the interactions between SALI and NSCLC. The shared genes of SALI and NSCLC regulate PBMC and ATII apoptosis to enhance SALI and are involved in cancer-related pathways that promote NSCLC development. SALI, sepsis acute lung injury; NSCLC, non-small-cell lung cancer.

In conclusion, the current study identified the shared signature genes between SALI and NSCLC (Figure 7). It was demonstrated that EZH2 plays a critical role in the pathogenetics of acute and chronic respiratory disease, the effect of which is related to the upregulation of PD-L1 expression. The inhibition of EZH2 could alleviate ATII apoptosis and decrease A549 proliferation, which might be a potential clinical treatment with which to modulate immune disorders.

## 6. Limitations

As with most studies, the design of the current study had some limitations. This study verified the key role of hub gene EZH2 identified by WGCNA in SALI and NSCLC in vitro but did not verify it in vivo. However, we demonstrated the diagnostic and prognostic value of EZH2 with some clinical data; thus, this limitation is not considered to have caused a large bias in the results of the study. We will continue to explore the underlying mechanism of EZH2 in SALI through animal studies.

## Data Availability

The existing datasets are available in a publicly accessible repository. Publicly available datasets were analyzed in this study. This data could be found here: GEO database (https://www.ncbi.nlm.nih.gov/gds/ (accessed on 1 August 2022)).
